# Clinicopathological Significance of Tumor Stem Cell Markers ALDH1 and CD133 in Colorectal Carcinoma

**DOI:** 10.30699/ijp.2020.127441.2389

**Published:** 2020-10-10

**Authors:** Maryam Rezaee, Elmira Gheytanchi, Zahra Madjd, Mitra Mehrazma

**Affiliations:** 1 *Oncopathology Research Center, Iran University of Medical Sciences, Tehran, Iran*; 2 *Department of Molecular Medicine, Faculty of Advanced Technologies in Medicine, Iran University of Medical Sciences, Tehran, Iran*; 3 *Department of Pathology, Hasheminejad Kidney Center, Iran University of Medical Sciences, Tehran, Iran*

**Keywords:** ALDH1, Cancer Stem Cells, CD133 Colorectal, cancer

## Abstract

**Background & Objective:**

Colorectal cancer (CRC) is the third most common cancer worldwide with a high mortality rate. The main causes of death in patients are recurrence and metastasis which are mainly attributed to the small subpopulation of cells within tumors called cancer stem cells (CSCs). This study aimed to evaluate the correlation between the expression of ALDH1 and CD133 as CSC associated markers and clinicopathological characteristics in CRC.

**Methods:**

In this cross-sectional study, a total of 483 CRC tumor samples were immunohistochemically stained for detection of CD133 and ALDH1 markers. Correlations of marker expression with clinicopathological factors were also evaluated.

**Results:**

There was a significant correlation between the luminal intensity of CD133 and neural invasion (*P*=0.05) and between the cytoplasmic intensity of CD133 and metastasis (*P*=0.05). In terms of H-score, a positive significant relation was observed between cytoplasmic expression of CD133 and lymph node (*P*=0.02), neural (*P*=0.04) and vascular invasion (*P*=0.02). The ALDH1 cytoplasmic expression showed a significant correlation with tumor size (*P*=0.001).

**Conclusion:**

Our findings showed that increased expression of CD133 and ALDH1 is associated with tumor progression and worse outcomes in CRC patients. These markers can be good candidates for localized targeting of CSCs using antibodies. Future researches need to be improved approaches for early detection of CRC, and treatment monitoring for CRC and other cancers.

## Introduction

Colorectal cancer is one of the third most prevalent cancers and the fourth cause of cancer-related mortality worldwide with 700,000 deaths annually (1-4). In terms of gender, CRC is considered as the second most common cancer in females and the third in males (5). The risk factors are related to habits or personal characteristics including age, history of chronic disease and lifestyle which are associated with CRC progression (3). Different mutations targeting genes such as tumor suppressors, DNA repairing, and oncogenes lead to CRC (6). Based on the gene mutation site, CRC could be categorized as sporadic (70%); inherited (5%) and familial (25%) (7). The chromosomal instability (CIN), microsatellite instability (MSI) and CpG island methylator phenotype (CIMP) has been reported as being the main mechanisms involved in CRC pathogenesis (8). The main pathways such as WNT, TP53, MAPK/PI3K, TGF-β, and mutated genes of *PTEN*, *PIK3CA*, C-MYC, *SMAD2, SMAD4,* *BRAF* and *KRAS* are affected by chromosomal changes and translocations (9-12). Despite the improvement in CRC diagnosis and therapy, the survival rate of patients with CRC still remains poor because of its drug resistance, metastasis, and recurrence (13, 14). Therefore, it is essential to develop and implement precise and suitable biomarkers to enhance diagnostic processes aiding clinicians in the detection of CRC in the earliest stages. Emerging evidence has shown that a subpopulation of cells also called tumor initiating or cancer stem cells (CSCs) with multi-potency and self-renewal characteristics have a critical role in CRC pathophysiology (15-17). They display chemotherapy resistance, differentiation potential, and high tumorigenicity and could be a promising therapeutic approach for CRC management (18, 19). CSCs has been effectively recognized in different solid tumors including CRC (20). Targeting of CSCs could be achieved in CRC through the various cell surface markers associated with self-renewal, including CD133, CD166, CD44, CD24, beta1 integrin-CD29, Lgr5, EpCAM (ESA), ALDH-1, Msi-1, DCAMLK1 or EphB receptors (18, 21). Hence, understanding which of these markers has the greatest effect on a patient’s diagnosis and prognosis has been the focus of many studies. The CD133 and ALDH-1 are among the main markers that have been linked to CSCs in CRC (22).

The cell surface marker of CD133 also known as AC133 in humans or prominin-1 in rodents is a five domain transmembrane molecule with a molecular weight of 120 kDa that has been identified as a normal and putative CSC marker in several cancers, including brain tumors, prostate carcinoma and CRC (23-29). Previous studies reported the *in-vitro* and *in-vivo* self-renewal and high tumorigenicity potential of CD133 in CRC (28, 30). It has been demonstrated that the CD133 expression in combination with other putative CSC markers correlated with clinical outcomes in CRC patients (31). 

Aldehyde dehydrogenase (ALDH) with several isoforms and different cellular functions and tissue distribution is located on chromosome 12 (30, 32). It is considered as a detoxifying enzyme for oxidation of intracellular aldehydes (32, 33). Isoforms of ALDH including ALDH-1 may lead to poor prognosis in patients and have been reported to be a putative stem cell marker in several cancers such as breast cancer (34), pancreatic adenocarcinoma (35), ovarian cancer (32) lung cancer and colorectal cancer (30, 32, 36). Resistance to chemotherapy is attributed to the transcriptional triggering in ALDH1 leading to drug and radiation resistance in CSCs which was first detected in hematopoietic progenitor cells (32). The role of ALDH-1 and its isoforms in CSCs characteristics including self-renewal, differentiation and epithelial-mesenchymal transition (EMT) has also been shown in xenotransplants of breast cancer and colon cancer (32, 37, 38). Previous studies in the literature indicated the prognostic value of ALDH1 expression in different cancers including CRC (39, 40) and some of them showed that there was any relation between the ALDH1 expression and tumor stage (41). 

Considering the lack of comprehensive information focusing on the relationship between concurrent expression of putative CSC markers ALDH1 and CD133 with main clinicopathological factors in CRC, current study was aimed to evaluate the immunohistochemical expression of these markers in CRC patients.

##  Materials and Methods

**Patient’s Data Collection and Sample Preparation**

In this cross-sectional study, formalin- fixed-paraffin-embedded (FFPE) tissues from 483 patients, who were diagnosed with CRC, were collected from three University-based Referral Centers of Hasheminejad, Rasoul-Akram and Firoozgar in Tehran, Iran, from 2009 to 2015. Patients’ medical records were evaluated to ensure the following demographic and clinicopathological parameters were included, those being ,age, gender, tumor size, grade, and stage, tumor location, tumor differentiation status, metastasis, lymph node and neural tissue invasion. All tumor histology was assessed using the hematoxylin and eosin (H&E) stained slides of tumors by experienced pathologists. Furthermore, patient data was kept confidential and the ethical use of patients’tissue samples was approved by the Ethics Committee of the Iran University of Medical Sciences (IR.IUMS.REC 1395.9311100010).

**Construction of Tissue Microarray (TMA) Blocks**

The CRC TMA blocks were prepared as aforementioned (40). In brief, the selection of tumor representative areas was performed from H&E slides for TMA preparation. The cores with a 0.6 mm diameter from marked tumor areas were punched into a tissue microarray block by Tissue Arrayer Minicore (ALPHELYS, Plaisir, France). The TMAs were constructed in five copies for each sample, the mean scoring of cores was then considered as the final score (27). Finally, the sectioning of TMA blocks was performed for immunohistochemical staining.

**Immunohistochemical (IHC) Determination of Markers**

The expression level of ALDH1 and CD133 was evaluated in CRC sections by the IHC method, as described previously (30). In sum, paraffin-embedded human CRC TMA blocks were sectioned at 5-μm thickness and then mounted onto Super frost slides (Superfrost plus, Thermo Scientific, Germany). All slides were dewaxed at 60^o^C for 30 min, deparaffinized with xylene and rehydrated in ethylic alcohol serial dilution and then treated with 3% hydrogen peroxide for 20 min. For antigenic epitope demasking the antigen retrieval process was done using pressurecooking system by submerging in citrate buffer (pH: 6.0) as target retrieval solution of both markers. After cooling the slides at room temperature, they were rinsed in Tris-buffered saline (TBS) three times. For primary staining of both markers, slides were respectively incubated with rabbit recombinant monoclonal ALDH1A1 antibody (1:200, overnight at 40^o^C, ab52492; Abcam; USA) and rabbit polyclonal CD133 antibody (1:250, overnight at 40^o^C, ab19898; Abcam; USA). Secondary staining was performed using EnVision TM/HRP, Rabbit/Mouse reagent (Dako; code K5007; Denmark; Ready to-use) for 30 min in a wet box at 37^o^C and then visualized by Dako REALTM DAB+ Chromogen (Dako; Denmark) based on the manufacturer’s instructions. After washing in TBS, slides were finally counterstained with Mayer’s hematoxylin dye (Dako; Denmark) for 15 min, and dehydrated in ethylic alcohol serial dilution and cleared in xylene.

**Immunostaining Evaluation and Scoring System**

Imaging analysis of the CRC TMA cores for ALDH1 and CD133 expression was performed in a coded manner independently by two pathologists (M.M. and M.R.) without knowledge about the patients' clinicopathological characteristics. Discrepancies between them were resolved by consensus. The staining percentage of cytoplasmic and luminal expression of CD133 and ALDH1, respectively, scored as follows: 1; less than 25%, score 2; 26-50%, score 3; 51-75% and score 4; more than 75%. The staining intensity was evaluated using a semi-quantitative system, ranging from non-stained to strong: 0; non-staining, 1; weakly staining, 2; moderate staining, 3; strong staining. The histochemical score results were finally calculated by multiplying the intensity in total percentage of positive cells ranging from 0-300 including less than 100, between 100-200 and more than 200.

**Statistical Analysis**

All statistical analyses were performed using SPSS version 22 (SPSS Inc., Chicago, IL., USA). Differences in ALDH1 and CD133, based on different clinicopathological parameters, were estimated by a χ2 test or Fisher’s exact test, if appropriate. The statistically significant difference was considered as P-value<0.05 and all statistics are presented to two decimal places.

## Results

**Clinicopathological and Tumor Characteristics of Patients**

In the present cross-sectional study, a total of 483 patients, diagnosed with colorectal adenocarcinoma, were included in this study. Out of 483 cases, 52.4% of cases were male and 47.6% of them were female with a mean age of 59.12±14.8 (range 23-92 years) at the time of diagnosis. Of all patients, 46.2% were ≥60 years old and 53.8% were <60 years old. The pathological results showed that the most prevalent tumor location was sigmoid (164 cases, 35.5%), and rectum (129 cases, 28%). The mean size of tumors was 5 cm, 324 (67.1%) tumors were less than 5 cm and 159 (32.9%) were more than 5 cm. Tumor cells were classified as well, moderately and poorly differentiated. Out of all tumor samples, there were 173 (35.8%) welldifferentiated, 277 (57.3%) moderately differentiated and 33 (6.8%) poorly differentiated adenocarcinoma cells. Thirty-four (7%) patients showed metastasis and 449 (93%) cases had no metastasis. Lymph node involvement was present in 181 patients (37.5%) and absent in 302 patients (62.5%). Neural invasion was observed in 97 (20.1%) cases and non-invasion in 386 (79.9%) cases and 74 (15.3%) cases had vascular invasion. There were 84 (17.4%) TNM stage 1, 208 (43.1%) TNM stage 2, 168 (34.8%) TNM stage 3 and 23 (4.8%) TNM stage 4 patients. The highest prevalence of tumor stage was 2a (38%).

**Correlation Between the Positive Expression of CD133 and CRC Clinicopathological Features**

**Fig. 1.**
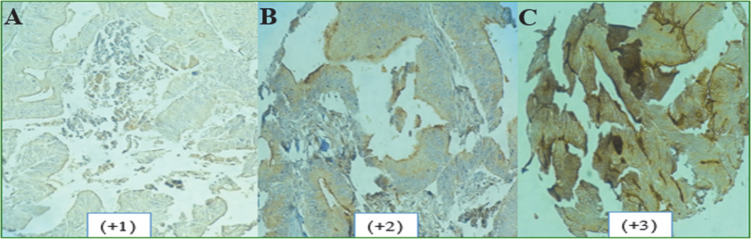
Expression patterns of CD133 in CRC tissues. (A) Weak; (B) Moderate; and (C) Strong intensity of staining

 The CD133 was expressed in cytoplasm of 358 and luminal area of 315 tumor samples and intensity of staining in CRC tissues are shown in Figure 1. A number of tumor samples were excluded from the study due to the tissue handling process or lack of tumor within the cores. The expression of CD133 varied considerably between different tumor samples. In terms of expression intensity, strong luminal intensity was observed in 75.9% of samples, and 42.7% of cases showed strong cytoplasmic intensity.

As indicated in Table 1, overall luminal and cytoplasmic expression of CD133 was not significantly associated with gender, age, tumor size (*P*>0.05). 66.1% of samples which showed neural invasion had high expression of CD133. There was significant correlation between the luminal intensity of CD133 expression and neural invasion (*P*=0.05). High expression of CD133 was found in 79.4% of tumor samples with lymph node involvement. However, no significant correlation was identified between strong luminal and cytoplasmic expression of CD133 and lymph node involvement. 40% of patients with metastasis showed high cytoplasmic expression of CD133 compared to 66.7% of metastatic cases with high luminal expression which was significant (*P*=0.05). A higher CD133 luminal intensity was detected in 76.4% of moderately or poorly differentiated malignant tumor cells compared with the well (75%) differentiated tumor cells. These values were relatively low in cytoplasmic expression of CD133. There was no significant association between luminal and cytoplasmic CD133 expression and tumor differentiation.

In terms of CD133 expression H-score as indicated in Table 1, 64.6% of male and 69.3% of female cases showed high H-score (>200) of luminal CD133 expression compared to 33.2% male and 26.8% female in cytoplasmic expression of CD133. 64.7. % of cases with H-score higher than 200 had tumor size more than 5cm and 55% of tumors with vascular invasion had higher H-score (>200) and 55.4% of tumors with neural invasion showed H-score higher than 200. The lymph nodes were involved in 73 (68.2%) cases with higher H-score. There was no significant association between the luminal and cytoplasmic H-score of CD133 with age, gender, tumor size, and metastasis (P>0.05). The luminal H-score of CD133 presented marked correlation with neural invasion (P=0.04). There was a significant association between the cytoplasmic H-score of CD133 with clinical factors including lymph node (*P*=0.02), neural (*P*=0.04), and vascular (*P*=0.02) invasion.

**Table 1:** Correlation between intensity and H-score of CD133 expression and clinicopathological characteristics in CRC

Patients and tumor characteristics	H-score of CD133 expression No. (%)		P-value	Intensity of CD133 expression				P-value
Expression Pattern	Low	High		Negative	Weak	Moderate	Strong	
Cytoplasmic	250(69.8)	108(30.2)		3(0.8)	77(21.5)	125(34.9)	153(42.7)	
Luminal	105(33.3)	210(66.7)		8(2.5)	25(7.9)	43(13.7)	239(75.9)	
Age ≥60 Cytoplasmic Luminal <60 Cytoplasmic Luminal	130(69.1) 55(32) 120(70.6) 50(35)	58(30.9) 117(68) 50(29.4) 93(65)	0.76 0.57	1(0.5) 4(2.3) 2(1.2) 4(2.8)	39(20.7) 13(7.6) 38(22.4) 12(8.4)	68(36.2) 21(12.2) 57(33.5) 22(15.4)	80(42.6) 134(77.9) 73(42.9) 105(73.4)	0.86 0.82
Gender Male Cytoplasmic Luminal Female Cytoplasmic Luminal	127(66.8) 62(35.4) 123(73.2) 43(30.7)	63(33.2) 113(64.6) 45(26.8) 97(69.3)	0.19 0.37	1(0.5) 5(2.9) 2(1.2) 3(2.1)	44(23.2) 16(9.1) 33(19.6) 9(6.4)	60(31.6) 26(14.9) 65(38.7) 17(12.1)	85(44.7) 128(73.1) 68(40.5) 111(79.3)	0.44 0.64
Tumor size ≤5 Cytoplasmic Luminal >5 Cytoplasmic Luminal	164(68.3) 69(32.4) 86(72.9) 36(35.3)	76(31.7) 144(67.6) 32(27.1) 66(64.7)	0.60 0.71	1(0.4) 5(2.3) 2(1.7) 3(2.9)	51(21.3) 17(8) 26(22) 8(7.8)	82(34.2) 25(11.7) 43(36.4) 18(17.6)	106(44.2) 166(77.9) 47(39.8) 73(71.6)	0.56 0.52
Vascular invasion Yes Cytoplasmic Luminal No Cytoplasmic Luminal	41(83.7) 18(45) 209(67.6) 87(31.6)	8(16.3) 22(55) 100(32.4) 188(68.4)	**0.02** 0.09	1(2) 2(5) 2(0.6) 6(2.2)	14(28.6) 2(5) 63(20.4) 23(8.4)	20(40.8) 8(20) 105(34) 35(12.7)	14(28.6) 28(70) 139(45) 211(76.7)	0.13 0.36
Neural invasion Yes Cytoplasmic Luminal No Cytoplasmic Luminal	53(72.6) 25(44.6) 190(57.4) 80(30.9)	17(23.3) 31(55.4) 104(31.4) 179(69.1)	**0.04** **0.04**	1(1.5) 1(1.8) 2(0.7) 7(2.7)	14(21.2) 4(7.1) 63(21.6) 21(8.1)	31(47) 14(25) 94(32.2) 29(11.2)	20(30.3) 37(66.1) 133(45.5) 202(78)	0.08 **0.05**
Lymph node involvement Yes Cytoplasmic Luminal No Cytoplasmic Luminal	53(80.3) 34(31.8) 197(67.5) 18(34.1)	13(19.7) 73(68.2) 95(32.5) 137(65.9)	**0.02** 0.67	1(0.8) 3(2.8) 2(0.9) 5(2.4)	31(23.8) 7(6.5) 46(20.2) 18(8.7)	49(37.7) 12(11.2) 76(33.3) 31(14.9)	49(37.7) 85(79.4) 104(45.6) 154(74)	0.53 0.70
Metastasis Yes Cytoplasmic Luminal No Cytoplasmic Luminal	18(72) 9(42.9) 232(69.7) 96(32.7)	7(28) 12(57.1) 101(30.3) 198(67.3)	0.80 0.33	1(4) 1(4.8) 2(0.6) 7(2.4)	9(36) 4(19) 68(20.4) 21(7.1)	5(20) 2(9.5) 120(36) 41(13.9)	10(40) 14(66.7) 143(42.9) 225(76.5)	**0.05** 0.21
Differentiation Cytoplasmic Well Moderate & Poor Luminal Well Moderate & Poor	97(71.9) 153(68.6) 44(36.7) 61(31.3)	38(28.1) 70(31.4) 76(63.3) 134(68.7)	0.51 0.32	0(0) 3(1.3) 1(0.8) 7(3.6)	31(23) 46(20.6) 14(11.7) 11(5.6)	43(31.9) 82(36.8) 15(12.5) 28(14.4)	61(45.2) 92(41.3) 90(75) 149(76.4)	0.51 0.12
Stage Cytoplasmic I & II III & IV Luminal I & II III & IV	148(67.6) 102(73.4) 69(34.3) 36(31.6)	71(32.4) 37(26.6) 132(60.7) 78(68.4)	0.24 0.61	2(0.9) 1(0.7) 6(3) 2(1.8)	46(21) 31(22.3) 18(9) 7(6.1)	71(32.4) 54(38.8) 28(13.9) 15(13.2)	100(45.7) 53(38.1) 149(74.1) 90(78.9)	0.52 0.70

*The values are shown in bold are statistically significant.

**Correlation Between the Positive Expression of ALDH1 and CRC Clinicopathological Features**

**Fig. 2:**
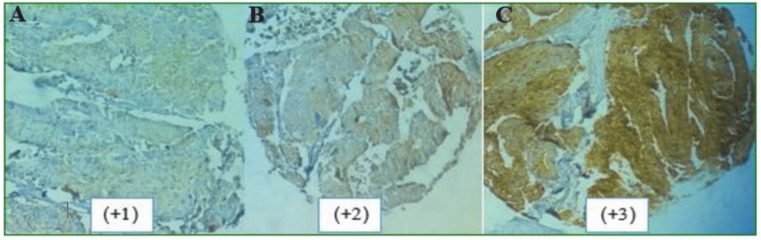
Expression patterns of ALDH1 in CRC tissues. (A) Weak (B) Moderate; and (C) Strong intensity of staining

The expression pattern of ALDH1 was mainly cytoplasmic in 40% (167/416) of TMA tumors (Figure 2), whereas 60% of cases showed negative intensity of staining. High ALDH1 expression was distinguished in 4 (2.7%) of the welldifferentiated tumors, 3 (1.3%) of moderately differentiated tumors and none of the highly expressed tumors showed poorly differentiated pattern. Seven cases with metastasis showed high ALDH1 expression. Only 1.3%, 2.1% and 1.7% of tumors with high intensity of staining developed lymph nodes, neural and vascular invasion, respectively (Table 2). The ALDH1 expression (intensity and H-score) was not significantly associated with age, gender, lymph nodes, and vascular invasion (*P*>0.05). Tumors more than 5cm in size exhibited H-score higher than 200 in 22.5% of cases (Table 2). A considerable positive correlation was found between the tumor size and H-score of ALDH1 expression in tumor cells (*P*=0.001). A marginal trend correlation was also observed between the neural invasion and ALDH1 intensity of staining (*P*=0.07). 

**Table 2:** Correlation between intensity and H-score of ALDH1 expression and clinicopathological characteristics in CRC

Patients and tumor characteristics	H-score of ALDH1 expression No. (%)			*P*	Intensity of ALDH1 expression No. (%)				*P*
**Expression Pattern**	**<100**	**100-200**	**<200**		**Negative**	**Weak**	**Moderate**	**Strong**	
Cytoplasmic	405(96.9)	11(2.6)	2(0.5)		249(60)	126(30)	34(8)	7(2)	
**Gender** **Male** Cytoplasmic **Female** Cytoplasmic	207(95.4) 196(98.5)	9(4.1) 2(1)	1(0.5) 1(0.5)	0.09	126(58) 123(61)	69(32) 57(29)	17(8) 17(8)	4(2) 3(2)	0.88
**Age** **23-40** Cytoplasmic **41-60** Cytoplasmic **>60** Cytoplasmic	40(95.2) 170(98.3) 195(96.1)	2(4.8) 3(1.7) 6(3)	0(0) 0(0) 2(1)	0.44	19(45.2) 100(57.8) 130(64.7)	17(40.5) 56(32.4) 53(26.4)	4(9.5) 16(9.2) 14(7)	2(4.8) 1(0.6) 4(2)	0.11
**Tumor size** **≤5** Cytoplasmic **>5** Cytoplasmic	276(95.8) 129(93.3)	2(3.4) 9(5.9)	1 (0.8) 1(0.8)	**0.001**	163(59.4) 82(60.7)	88(32) 37(27.4)	20(7.3) 13(9.6)	4(1.5) 3(2.2)	0.66
**Vascular invasion** **Yes** Cytoplasmic **No** Cytoplasmic	56(96.6) 339(96.9)	2(3.4) 9(2.6)	0(0) 2(0.6)	0.75	36(62.1) 207(59.5)	15(25.9) 107(30.7)	6(10.3) 28(8)	1(1.7) 6(1.7)	0.85
**Neural invasion** **Yes** Cytoplasmic **No** Cytoplasmic	79(98.6) 326(96.4)	1(1.4) 10 (3)	0(0) 2(0.6)	0.79	53(72.6) 190(57.4)	17(23.3) 104(31.4)	3(44.1) 30(9.1)	0(0) 7(2.1)	0.07
**Lymph node involvement** **Yes** Cytoplasmic **No** Cytoplasmic	82(97.1) 256(96.6)	3(2.1) 7(2.6)	1 (0.8) 1(0.8)	0.88	82(54.3) 166(26.9)	53(35.1) 73(27.7)	14(9.3) 20(7.6)	2(1.3) 5(1.9)	0.32
**Metastasis** **Yes** Cytoplasmic **No** Cytoplasmic	31(95.8) 374(96.8)	1(4.2) 10(2.6)	0(0) 2(0.5)	0.55	16(66.7) 225(59.8)	7(29.2) 122(29.8)	1(4.2) 32(8.5)	0(0) 7(1.9)	0.93
**Differentiation** **Cytoplasmic** Well Moderate Poor	140(94.6) 237(97.9) 28(100)	7(4.8) 4(1.7) 0(0)	1(0.7) 1(0.4) 0(0)	0.33	82(56.2) 145(61.2) 18(66.7)	45(30.8) 73(30.8) 6(22.2)	15(10.3) 16(6.8) 3(11.1)	4(2.7) 3(1.3) 0(0)	0.61

*The values are shown in bold are statistically significant.

**Correlation Between the Expression of CD133/ALDH1 and CRC Clinicopathological Features**

The association between immunohistochemical CD133 and ALDH1 expression was measured in the 283 matched cases using Pearson’s chi-square. A significant reciprocal relation was found between the CD133 and ALDH1 expression (*P*<0.001). Based on the combined analysis, the CD133/ALDH1 expression was divided into 4 phenotypes, including CD133-High/ALDH1-High (8.8%) indicating high expression of both markers, CD133-Low/ALDH1-Low (8.8%) indicating low expression of both markers.CD133-High/ALDH1-Low (81.3%), and CD133-Low/ALDH1-High (1.1%). The correlation between CD133/ALDH1 phenotypes and clinicopathological characteristics of CRC patients was evaluated using the one-way ANOVA and Tukey’s post hoc analysis (Table 3). A marked positive correlation was only observed between the CD133-High/ALDH1-Low phenotype and neural invasion representing that this phenotype was more dominant in CRC patients compared to other phenotypes (*P*=0.037).

**Table 3:** Correlation between CD133/ALDH1 phenotypes and clinicopathological characteristics in CRC

Patients and tumor characteristics	CD133/ALDH1 phenotypes				*P*
	CD133High/ ALDH1High	CD133Low/ALDH1Low	CD133High/ ALDH1Low	CD133Low/ ALDH1High	
Gender **Male** **Female**	13(8.3) 12(9.4)	17(10.9) 8(6.3)	125(80.1) 105(82.7)	1(0.6) 2(1.6)	0.49
Age **≤60** **>60**	14(9.4) 11(8.2)	13(8.7) 12(9)	121(81.2) 109(81.3)	1(0.7) 2(1.5)	0.90
Tumor size **≤5** **>5**	14(7.3) 11(12.2)	16(8.3) 9(10)	160(82.9) 70(77.8)	3(1.6) 0(0)	0.32
Vascular invasion **Yes** **No**	4(12.1) 21(8.4)	2(6.1) 23(9.2)	27(81.8) 203(81.2)	0(0) 3(1.2)	0.76
Neural invasion **Yes** **No**	0(0) 25(10.5)	1(2.3) 24(10.1)	42(95.5) 188(78.6)	1(2.3) 2(0.8)	**0.03**
Lymph node involvement **Yes** **No**	9(9.4) 16(8.6)	7(7.3) 18(9.6)	80(83.3) 150(80.2)	0(0) 3(1.6)	0.56
Metastasis **Yes** **No**	1(5.6) 24(9.1)	2(11.1) 23(8.7)	15(83.3) 215(81.1)	0(0) 3(1.1)	0.90
Differentiation **Well** **Moderate & Poor**	11(10.2) 14(8)	9(8.3) 16(9.1)	85(78.7) 145(82.9)	3(2.8) 0(0)	0.14

*The values shown in bold are statistically significant

## Discussion

Colorectal cancer is one of the commonest visceral cancers and a leading cause of death throughout the world (27). Despite the improving trend of diagnostic and therapeutic processes, the majority of CRC patients experience a poor prognostic disease which is often manifested by drug resistance, recurrence, and metastasis (30). It is believed that cancers may be gradually organized by their own CSCs as a rare and small sub-population of cancer cells with potential to cause metastasis and recurrence (42-45). Several studies have been performed in this regard to identify specific markers for CRC CSCs but there is still controversy over the special marker to distinguish a distinct CSC population (12, 18, 32, 46). Therefore, discovery of more precise CSC markers would be helpful in early diagnosis, prognostic classification and well-organized targeted therapy of CRC and for improving prognosis through the metastasis and local recurrence prevention. The current study was carried out to assess the expression pattern of CD133 and ALDH1 as putative cancer stem cell markers in CRC using TMA and IHC techniques among Iranian population for the first time. We showed the increased expression of CD133 in the majority of CRC tumor samples compared to ALDH1. Lymph node, vascular, and neural invasion were more common in cases with higher CD133 expression than in cases with negative or low CD133 expression, which were statistically significant (P<0.05). In line with our findings, a meta-analysis based on the 37 studies reported increased CD133 expression in CRC as a poor prognostic factor in CRC patients and it was positively correlated with lymphatic and vascular invasion, distant metastasis, and tumor T category (47). Li and colleagues demonstrated that an increased percentage of CD133 positive tumor cells was linked with poor prognosis in CRC patients with higher stage (48). We observed CD133 overexpression in CRC cases with advanced stages (III & IV) but differences were not statistically significant (*P*>0.05). In contrast, another study reported the correlation between early stage (stage I) and worse outcome in CRC patients as a robust predictor factor (49).

Regarding CD133 expression patterns, immunohistochemical technique has been applied in various studies to show that the CD133 was mainly expressed on luminal surface and cell membrane of tumors (47-54), while other studies detected that it could be expressed both on cytoplasm and membrane of tumor cells (44, 55). Differences in the clinicopathological significance of tumor stem cell markers including CD133 is largely affected by the expression patterns of these markers. Epithelial to mesenchymal transition (EMT) and finally an invasive and metastatic phenotype is considerably linked to the cytoplasmic to membranous shift in CD133 localization (49). The CD133 expression at luminal surface of CRC tumor glands has been reported as an independent predictive marker of CRC (55). Our IHC findings revealed that the CD133 was mainly located at the luminal surface of most tumor samples (66.7% as H-score and 75.9% as intensity of staining) which were significantly correlated with CRC neural invasion (*P*<0.05). Kojima* et al. *reported the luminal and cytoplasmic expression of CD133 in colorectal cancer mainly in well and moderately differentiated tumors but not in the poorly differentiated form which is significantly correlated with distant metastasis (56). We observed the expression of CD133 not only in well and moderately differentiated tumors but also in poorly differentiated cells that is correlated with metastasis (*P*<0.05). These results are consistent with Horst et al.’s study, who found CD133 expression at the luminal surface of CRC gland with lumina shedding as a predictive marker of poor prognosis (57).

ALDH1 is a kind of detoxifying enzyme and potential CSC markers have been recognized in different cancers including head and neck (30) and breast (58) and several studies has focused on its prognostic significance in different cancers (30). An increase in cell proliferation and invasion capacity has been attributed to the role of ALDH1 in CSC characteristics and in the biological features of tumors (58, 59). Zhou and colleagues have demonstrated the relation between high expression of ALDH1 and poor outcome in CRC patients but it was not correlated to Lymph node invasion compared to CD133 (59). Lugli *et al.* reported no correlation between the ALDH1 expression and survival (59). They detected high ALDH1 expression in only 23.3% of CRC cases without any differences in survival rate (30). Although a large number of studies have investigated the role of ALDH1 expression on CRC patient’s outcomes, controversy regarding prognostic significance of ALDH1 still remains. Therefore, further research should be conducted to reach a definite conclusion. Our study indicated that the ALDH1 CSC marker was only overexpressed in 40% of CRC patients which are partly consistent with the findings of the Lugli study (60). Further significant findings of the present study was that well and moderately differentiated tumors had smaller tumor size and exhibited a lower expression of ALDH1 in terms of H-score compared with poorly differentiated tumors which was statistically significant (*P*=0.001). Meanwhile, a meta-analysis study on the prognostic value of ALDH1, as a cancer stem cell marker indicated that ALDH1 was expressed in different levels among various populations of CRC patients (18). Nevertheless, differences between expression degrees of CSC markers and their complicated mechanisms are not yet clear. Liu* et al. *(2014) supposed that there were differences in expression patterns of ALDH1 and its prognostic significance between western and eastern people (61). Chen and colleagues systematically reviewed a large population of patients from western and eastern countries and concluded that the expression rate of ALDH1 is higher among the western populations (52%) compared to low rate expression among the eastern populations (39%). 

Since CRC cells expressing one of putative CSC markers displayed high tumorigenicity especially in combination with other CSC markers (62), the assessment of combined markers may be helpful in better understanding tumor characteristics than those considered individually. Co-expression of CD133 and ALDH1 has been seen in a wide range of tumor cells, including lung (61) and gastric cancers which are involved in tumor invasion, metastasis and poor prognosis of patients (62). Therefore, we assessed the prognostic significance of combined CD133/ALDH1 phenotypes with clinicopathological factors in CRC. Our analysis revealed that CD133High/ALDH1Low phenotype was more frequent in CRC tumors than other combined phenotypes. There was also a positive significant correlation between this phenotype with neural invasion of tumors. Considering the different degree of CSC markers expression in several malignancies and differences among the various populations and markers localization, it can be concluded that CD133High/ALDH1Low phenotype may confer tumor progression behavior in CRC patients.

## Conclusion

Combined detection of CD133 and ALDH1, as CSC associated markers, is likely to be valuable in understanding their clinicopathological and prognostic significance in CRC. Furthermore, patient classification in combined expression phenotypes of putative CSC markers could be identified as appropriate targeted therapies according to different subgroups of CRC patients and thus management of tumor progression, especially in advanced cases.
